# Effects of standardized hydro-alcoholic extract of *Vaccinium arctostaphylos* leaf on hypertension and biochemical parameters in hypertensive hyperlipidemic type 2 diabetic patients: a randomized, double-blind and placebo-controlled clinical trial

**Published:** 2019

**Authors:** Reza Mohtashami, Hasan Fallah Huseini, Farzaneh Nabati, Reza Hajiaghaee, Saeed Kianbakht

**Affiliations:** 1 *Medicine, Quran and Hadith Research Center, Baqiyatallah University of Medical Sciences, Tehran, Iran *; 2 *Medicinal Plants Research Center, Institute of Medicinal Plants, ACECR, Karaj, Iran*

**Keywords:** Vaccinium arctostaphylos, Hypertension, Diabetes mellitus, Dyslipidemia

## Abstract

**Objective:**

To study the blood pressure, lipid and glycemic effects and safety of *Vaccinium arctostaphylos *leaf in the hypertensive hyperlipidemic type 2 diabetic patients.

**Materials and Methods:**

The patients took 350 mg standardized plant leaf hydro-alcoholic extract capsule (n=50) or placebo capsule (n=50) three times daily alongside conventional drugs for 2 months. At the baseline and endpoint, systolic and diastolic blood pressures and blood levels of fasting glucose (FG), 2-hr postprandial glucose (2hPPG), glycosylated hemoglobin (HbA1c), total cholesterol (TC), LDL-C, triglyceride, HDL-C, SGOT, SGPT and creatinine were determined in both groups. To evaluate the extract safety, serum SGOT, SGPT and creatinine levels were tested; also, the patients were requested to report any adverse effects.

**Results:**

FG, 2hPPG, HbA1c, TC, LDL-C, triglyceride and systolic and diastolic blood pressures were decreased, whereas HDL-C was increased significantly in the extract group compared to those of the placebo group at the endpoint (for all cases, p<0.05). The extract did not significantly influence other parameters and no adverse effects were reported.

**Conclusion:**

*V. arctostaphylos* leaf hydro-alcoholic extract as an adjunct to the conventional drugs has additional antihypertensive as well as anti-dyslipidemic and anti-hyperglycemic effects in hypertensive hyperlipidemic type 2 diabetic patients. Besides, the extract lacks hepatic, renal and patient-reported adverse effects.

## Introduction

Hypertension, dyslipidemia and diabetes mellitus type 2 (DMT2) are common co-morbid risk factors for cardiovascular diseases (CVD) (Larsen et al., 2018 ▶; Katsiki et al., 2018 ▶). CVD are the number-one cause of death in the world, with more than 17.9 million deaths per year in 2015 (GBD 2015 ▶ Mortality and Causes of Death Collaborators, 2016), which is predicted to exceed 23.6 million by 2030 (Mozaffarian et al., 2018 ▶). Conventional drugs used in the management of hypertension, dyslipidemia and DMT2 have limited efficacy and safety (Larsen et al., 2018 ▶; Katsiki et al., 2018 ▶). Plants were the first source of therapeutics and still play an important role in drug discovery. Medicinal plants can be used as alternative or complementary agents in the treatment of cardiometabolic diseases (Chrysant and Chrysant, 2017 ▶).* Vaccinium arctostaphylos* L. (Caucasian whortleberry) (Ericaceae family) is the only species of the *Vaccinium *genus growing in Iran. *V. arctostaphylos* leaf and fruit have been used as anti-hypertensive and anti-diabetic agents in the Iranian folk medicine (Mozaffarian, 2013 ▶). Pharmacological studies have demonstrated that *V. arctostaphylos* has metabolic effects.* V. arctostaphylos* leaf aqueous extract had anti-hypertensive effect in the rat model of two-kidney, one-clip hypertension (Khalili et al., 2011 ▶). Also, the ethanolic extract of *V. arctostaphylos *fruit had anti-hyperglycemic, anti-hypertriglyceridemic and antioxidant effects, and it stimulated the expression of pancreatic insulin and myocardial glucose transporter 4 genes in alloxan-induced diabetic rats. Besides, it inhibited α-glucosidase activity, *in vitro* (Feshani et al., 2011 ▶). Moreover, *V. arctostaphylos *leaf methanolic extract and its constituent, quercetin demonstrated inhibitory effect on the activity of pancreatic α-amylase, *in vitro* (Nickavar and Amin, 2011 ▶). Daily intake of the *V. arctostaphylos* leaf hydro-alcoholic extract for 4 weeks by type 2 diabetic patients indicated anti-hyperglycemic and anti-inflammatory effects in a double-blind placebo-controlled clinical trial (Abidov et al., 2006 ▶). Daily administration of *V. arctostaphylos *fruit hydro-alcoholic extract to type 2 diabetic patients for 2 months, had anti-hyperglycemic effects in a randomized, double-blind and placebo-controlled clinical trial (Kianbakht et al., 2013 ▶). In a 4-week randomized, double-blind and placebo-controlled clinical trial on hypercholesterolemic and hypertriglyceridemic patients, daily administration of the *V. arctostaphylos *fruit hydro-alcoholic extract demonstrated anti-hypercholesterolemic, anti-hypertriglyceridemic and antioxidant effects, while it did not affect HDL-C levels (Soltani et al., 2014 ▶). In another randomized, double-blind and placebo-controlled clinical trial on hypercholesterolemic and hypertriglyceridemic patients, the *V. arctostaphylos *fruit hydro-alcoholic extract had anti-hypercholesterolemic, anti-hypertriglyceridemic effects and elevated HDL-C levels after 2 months of daily use (Kianbakht et al., 2014 ▶). However, the effect of *V. arctostaphylos* on blood pressure has not yet been investigated in humans. Therefore, the present trial was conducted to evaluate the possible anti-hypertensive effect of the plant. 

## Materials and Methods

**Plant material**

*V. arctostaphylos* was collected from Ardebil province, Iran in October 2012 and a botanist identified the plant. A voucher specimen of the plant (number 22110) was deposited in Tehran University Central Herbarium. The leaves were separated from the plant, washed and dried in shade at room temperature and thereupon, the dried leaves were ground into powder.

**Figure 1:**
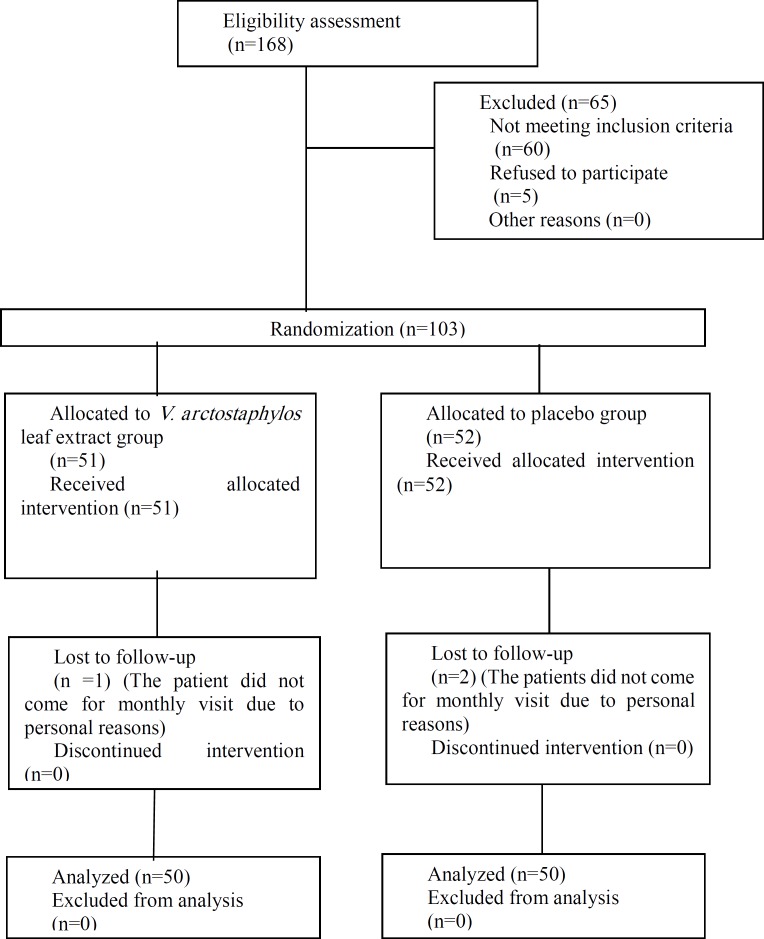
The CONSORT flowchart of the trial

**Extraction**

The dried leaf powder (20 kg) was extracted using 70% ethanol as the solvent in a percolator for 72 hr, the solvent was completely removed from the extract by a rotary evaporator; next, toast powder as an excipient was mixed with the extract and the mixture was ground into powder. Finally, 7.23 kg of the dry leaf extract powder was produced. The excipient constituted 15 percent of the final leaf extract weight. The extraction yield was 25%. 

**Preparation of the leaf extract and placebo capsules**

Gelatin capsules containing the extract powder as the drug and toast powder as the placebo, were separately prepared by a hand-operated capsule-filling machine (Scientific Instruments and Technology Corporation, USA). The *V. arctostaphylos* extract capsules contained 350 mg of the leaf extract powder. The *V. arctostaphylos *and placebo capsules were identical in all respects. 

**Phytochemical analyses of the leaf extract**

To standardize the extract, the total phenolic, flavonoid and anthocyanin contents were determined by spectrophotometry and the chlorogenic acid and quercetin contents were measured by HPLC as described previously (Gutfinger, 1981 ▶; Khonche et al., 2017 ▶; Iranian Herbal Pharmacopoeia Commission, 2002 ▶; Wen et al., 2012 ▶; Verma and Trehan, 2013 ▶).

**Determination of the total phenolic content**

The total phenolic contents were determined using the Folin-Ciocalteu colorimetric method (Gutfinger, 1981 ▶). In brief, the plant extract solution (1 mL) was mixed with 500 μL of the Folin-Ciocalteu reagent and 5 mL distilled water in a volumetric flask. After five minutes, 1 mL of 15% sodium carbonate solution was added to the mixture and kept in the dark for 30 minutes. Afterward, the absorbance was determined at 725 nm using a spectrophotometer (Human, USA). Gallic acid was used to generate the standard curve, and the reduction of the Folin-Ciocalteu reagent by the samples was expressed as mg of gallic acid equivalents per gram of extract. 

**Determination of the total flavonoid content**

The extract (1 mg/mL) or standard was mixed with 4 mL of distilled water and 300 μL of 5% sodium nitrite solution and after five minutes, 300 μL of 10% aluminum chloride solution was added to the mixture. After six minutes, 2 mL of 1 M sodium hydroxide and 3 mL of distilled water were added to the mixture. Then, the solution was properly mixed and absorbance was measured at 510 nm using a spectrophotometer (Human, USA). Rutin (100 μg/mL up to 1200 μg/mL) was used to construct the standard curve and the results were expressed as mg of rutin equivalents per gram of extract (Khonche et al., 2017 ▶).

**Determination of the total anthocyanin content**

The total anthocyanin content of the extract was measured using a spectrophotometric method (Iranian Herbal Pharmacopoeia Commission, 2002 ▶). Here, 1 g of the extract was dissolved in 15:85% v/v% of hydrochloric acid 1.5 N in 95% ethanol. The solution was filtered through a paper filter into a 250 mL volumetric flask and diluted with solvent to 250 mL. The absorbance of the solution was measured at 535 nm by a spectrophotometer (Human, USA). The total anthocyanin content per gram of extract was calculated using the following formula:

Milligrams of total anthocyanins per gram of extract= (Ab×25000)/982

Where Ab=absorption of solution.

**Determination of the chlorogenic acid content**

For this purpose, a Knauer HPLC (Germany) equipped with a K1001 pump and a K2501 UV detector (Knauer, Germany), was used. The mobile phase used in this study consisted of acetonitrile and 0.5% aqueous phosphoric acid (11.5:88.5% v/v%) and the flow rate was 1.0 mL/min. Also, determination wavelength was 327 nm and a Phenomenex NX-C18 analytical column was employed (diameter 4.6 mm and length 250 mm) (Wen et al., 2012 ▶). All reagents were of analytical grade and purchased from Merck (Germany). The result was given as mg of chlorogenic acid per gram of extract.

**Determination of the quercetin content**

The chromatographic separations were done using a YMC Triart C18 column (250 mm×4.6 mm i.d., 5 μm). A reverse phase HPLC assay was carried out using isocratic elution at a flow rate of 1 mL/min, a mobile phase of 35: 65% (acetonitrile: 0.2 phosphoric acid) and detection was done at 360 nm. The volume injection for each solution was 50 μL. The total run time was 16 min for each injection. Before injection into the HPLC, the solvents and distilled water were filtered through a PVDF (polyvinylidene fluoride) membrane filter using a set of glass bottles with the aid of a vacuum pump (Verma and Trehan). The result was expressed as mg of quercetin per gram of extract.

**The trial protocol**

A 2-arm, randomized, double-blind, placebo-controlled parallel-group trial was performed in Diabetes and Metabolic Diseases Clinic of Tehran University of Medical Sciences (Tehran, Iran) from April 21, 2013 to June 22, 2017. Inclusion criteria were as follows: Iranian male and female hypertensive hyperlipidemic type 2 diabetic outpatients; patients aged between 40 and 80 years old; patients with blood levels of HbA1c between 7% and 9%; fasting blood LDL-C levels of 100-150 mg/dl; systolic and diastolic blood pressure of 14-16 and 9-10 mmHg, respectively; patients taking 15 mg glibenclamide, 2 g metformin and 20 mg atorvastatin per day; patients taking three agents from different classes of antihypertensive drugs (i.e. thiazide, angiotensin converting enzyme inhibitor, angiotensin receptor antagonist, beta receptor antagonist, calcium channel blocker), daily. Exclusion criteria were as follows: Patients taking other drugs that may affect the blood glucose and lipid levels; patients with cardiac, renal, hepatic, and hematological diseases, hypothyroidism, tachycardia, vertigo and seizure; patients with a history of gallstones or gall bladder surgery; pregnant women; women planning pregnancy; and breast-feeding women. One hundred and sixty eight patients were screened and 65 patients were excluded. The remaining patients (n=103) were randomized to the leaf extract and placebo groups (Figure 1). Block randomization with computer-generated random number table and sequentially numbered containers each representing a block consisting of three patients, was used for the leaf extract /placebo allocation. The patients were instructed to take one leaf extract or placebo capsule three times a day alongside the standard treatments. Before intervention (i.e. baseline) and 2 months after intervention, blood pressure and biochemical parameters were evaluated by 24-hr ambulatory blood pressure monitoring and an auto-analyzer (Hitachi 917, Japan). Primary outcome variables included blood levels of HbA1c and LDL-C as well as systolic and diastolic blood pressures. Secondary outcome variables were blood levels of fasting glucose, 2 hr postprandial glucose (2hPPG), total cholesterol, triglyceride, HDL-C, creatinine, SGOT and SGPT. To evaluate the extract safety, serum levels of SGOT, SGPT and creatinine were determined and the patients were requested to report any adverse effects. Three different persons generated the random allocation sequence, enrolled the patients and assigned them to either leaf extract or placebo groups. These persons, care-providers and patients were blinded to interventions. Adherence to the regimen of extract and placebo capsules was monitored by counting returned drugs and asking about the number of capsules taken. The protocol was approved by the ethics committee of Endocrinology and Metabolism Research Institute of Tehran University of Medical Sciences (approval No. E-00104). The trial was performed in accordance with the revised Declaration of Helsinki 2013. The participants gave written informed consent before enrolment. The trial was registered (IRCT201701282288N11) in the Iranian Registry of Clinical Trials.

**Statistical analysis**

 Fifty patients in each group was the sample size calculated to detect 4 cmHg difference of systolic blood pressure numbers between the groups, considering type I error = 0.05 and 80% power. The chi-square and independent samples t tests were used for data analyses and a p<0.05 was considered significant. The data were analyzed by the intention-to-treat approach.

## Results

**Phytochemical analyses**

**Figure 2:**
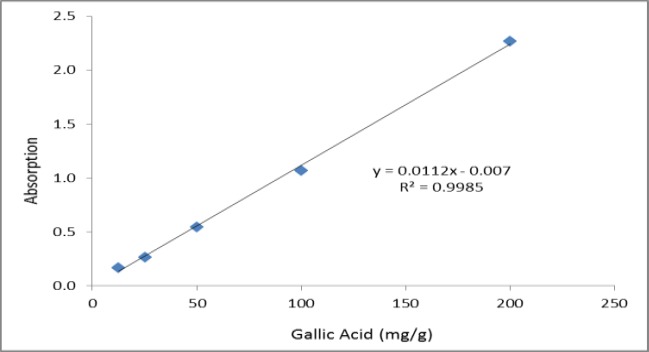
Calibration curve used for determination of the total phenolic content

**Figure 3:**
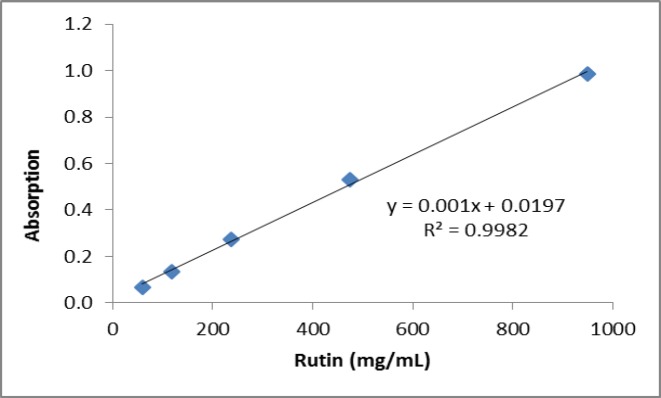
Calibration curve used for determination of the total flavonoid content

**Figure 4:**
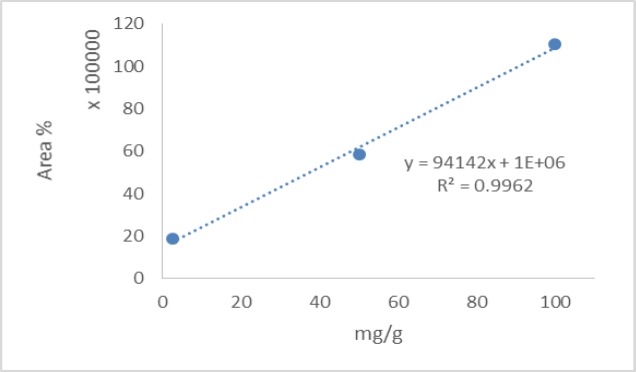
Calibration curve used for determination of the chlorogenic acid content

**Figure 5:**
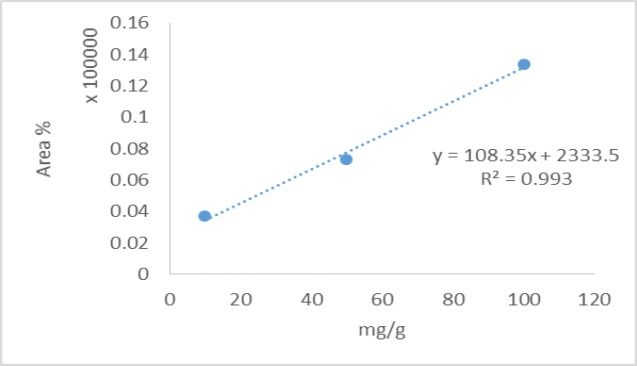
Calibration curve used for determination of the quercetin content

**Figure 6:**
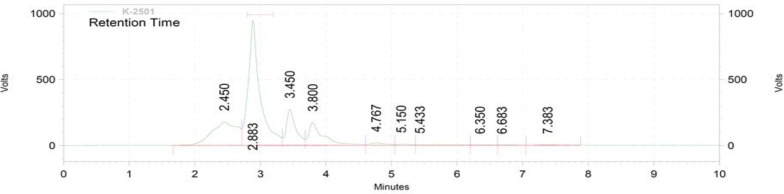
HPLC chromatogram showing the extract chlorogenic acid

**Figure 7:**
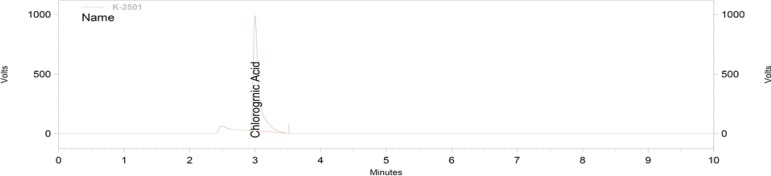
HPLC chromatogram showing the standard chlorogenic acid

**Figure 8:**
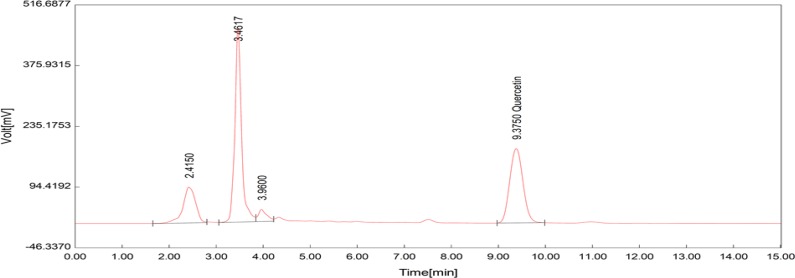
HPLC chromatogram showing the extract quercetin

**Figure 9:**
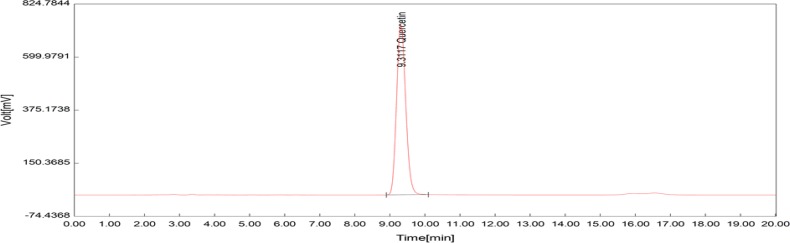
HPLC chromatogram showing the standard quercetin

The total phenolic content as milligrams of gallic acid equivalents per gram of the extract was 175±1.59 (Figure. 2). The total flavonoid content as milligrams of rutin equivalents per gram of extract was 340±0.8 (Figure 3). The total anthocyanin content per gram of the extract was 0.738±0.005 mg. Moreover, the chlorogenic acid and quercetin contents per gram of the extract were 97.15±0.9 and 22.36±0.5 mg, respectively (Figures 4-9). The values are expressed as mean of three measurements ± standard deviation.

**Clinical trial**

 Fifty patients in the extract group and Fifty patients in placebo group, completed the trial (Figure 1). The patients fully complied with the treatments. Demographics of the patients were not significantly different between the extract and placebo groups (Table 1). The groups did not differ significantly with regard to the baseline values of the outcome parameters and the types and doses of the antihypertensive drugs. The leaf extract significantly decreased systolic and diastolic blood pressures (both p<0.001) as well as blood levels of fasting glucose, 2 hrs postprandial glucose, HbA1c, total cholesterol, LDL-C and triglyceride and increased the HDL-C level compared to the placebo, at the endpoint (p<0.05 to p<0.001) (Tables 2 and 3). The extract did not significantly affect the blood levels of SGOT, SGPT and creatinine compared to the placebo at the end of the study (p=0.630, p=0.552, and p=0.674 respectively) (Table 3). Moreover, the patients did not report any adverse effects.

**Table 1:** Demographic characteristics of the study participants. Where appropriate, values are given as mean±standard deviation

**Parameter**	***V. arctostaphylos*** ** leaf extract group**	**Placebo group**
**Age (years)**	66±10.5	69.1±9
**Gender**	27 male and 23 female	29 male and 21 female
**Duration of diabetes mellitus type 2 (years)**	12.3±6.9	10.6±5.3
**Body mass index (kg/m** ^2^ **)**	30.1±10.3	29.8±7.8

**Table 2:** Blood pressure before and after intervention. Independent samples t test was used for statistical analysis

**Parameter**	**Mean ± SD before intervention **	**p-value (extract versus placebo)**	**Mean ± SD** **after intervention **	**p-value (extract versus placebo)**
**Systolic blood pressure (mmHg)**	150.8±6.7 L 151.7±5.4 P	0.511	127.3±10.5 L 151±7.9 P	<0.001
**Diastolic blood pressure (mmHg)**	88.9±13.9 L 90.8±6.5 P	0.436	71.5±8.9 L 88.1±7.7 P	<0.001

L: *V. arctostaphylos* leaf extract group; P: placebo group; and SD: standard deviation.

**Table 3:** Blood parameters before and after intervention. Independent samples t test was used for statistical analysis

**Parameter**	**Mean ± SD before intervention **	**p-value (extract versus placebo)**	**Mean ± SD** **after intervention **	**p-value (extract versus placebo)**
**Fasting glucose (mg/dl)**	195.7±40.1 L 189.7±39 P	0.197	139.0±25.7 L 201.6±45.6 P	<0.001
**2 hours postprandial glucose (mg/dl)**	279. 8±62.3 L 265. 1±47.9 P	0.104	220.5±45.7 L 285.0±66.3 P	0.011
**HbA1c (%)**	9±0.8 L 8.9±0.7 P	0.617	7.4±0.7 L 9±0.7 P	0.005
**Total** **Cholesterol (mg/dl)**	228.0±42.6 L 217.7±49.7 P	0.505	166.6±40.9 L 209.6±52.4 P	0.004
**Triglyceride (mg/dl)**	265.3±58.4 L 252.9±100.9P	0.503	167. 8±54.9 L 226.7±100 P	0.001
**LDL-C (mg/dl)**	132.4±26.7 L 124.9±24.7 P	0.200	95.3±20.4 L 140.7±26. 6 P	<0.001
**HDL-C (mg/dl)**	46.8±12.1 L 45.2±10.1 P	0.872	55.4±10.8 L 46.0±8.1 P	0.001
**SGOT (U/L)**	25.2±6.3 L 21.2±7.2 P	1.000	23. 2±12.6 L 24.3±12.2 P	0.630
**SGPT (U/L)**	15.9±8.9 L 17.2±6.9 P	0.478	14.8±10.2 L 16.5±9.0 P	0.552
**Creatinine (mg/dl) **	1.1±0.3 L 0. 8±0.1 P	0.205	1.0±0.1 L 1. 0±0.1 P	0.674

L: *V. arctostaphylos* leaf extract group; P: placebo group; and SD: standard deviation.

## Discussion

DMT2 is associated with 2 to 3-fold increased risk of cardiovascular (CV) events and CV etiology accounts for 80% of the mortality in DMT2. Hyperglycemia and other metabolic risk factors including hypertension and dyslipidemia are the major causes of initiation and progression of CVD in DMT2. This necessitates development of drugs which control the metabolic risk factors in DMT2 (Yandrapalli and Aronow, 2017 ▶; Lupsa and Inzucchi, 2018 ▶). 

The results of the present study show that the hydro-alcoholic extract of* V. arctostaphylos *leaf has anti-hypertensive effect and improves glycemic control and lipid profile in the hypertensive, hyperlipidemic type 2 diabetic patients without any hepatic, renal and patient-reported side effects. In other words, *V. arctostaphylos* leaf extract appears to be an effective and safe agent for the treatment of hypertension, hyperglycemia and dyslipidemia in DMT2. Therefore, *V. arctostaphylos* leaf extract may prevent CV events associated with DMT2. The results are in accordance with the previous research demonstrating the anti-hypertensive, anti-hyperglycemic, anti-hypercholesterolemic, anti-triglyceridemic and HDL-C raising effects of *V. arctostaphylos *in animal models and clinical trials (Abidov, 2006 ▶; Khalili et al., 2011 ▶; Feshani et al., 2011 ▶; Kianbakht et al., 2013 ▶; Kianbakht et al., 2014 ▶; Soltani et al., 2014 ▶). The phenolic compounds may have anti-hypertensive, anti-hyperglycemic and anti-dyslipidemic effects (Williamson, 2017 ▶; Haddad and Eid, 2017 ▶; Naveed et al., 2018 ▶). Therefore, the bioactive components identified in the *V. arctostaphylos *leaf extract in this study, i.e. phenolic compounds such as flavonoids, quercetin, chlorogenic acid and anthocyanins may be involved in the anti-hypertensive, anti-hyperglycemic and anti-dyslipidemic effects of the leaf extract of* V. arctostaphylos*. These compounds may decrease blood pressure through calcium antagonistic action, angiotensin converting enzyme inhibition and endothelial nitric oxide synthase activation. The anti-hyperglycemic action of these compounds may be mediated via increased expression of skeletal muscle glucose transporter 4, prevention of hepatic glycogenolysis by inhibition of the hepatic glucose-6-phosphatase and prevention of the intestinal glucose absorption by inhibition of glucose-6-phosphate translocase 1, α-amylase and α-glucosidase. Up-regulation of peroxisome proliferator-activated receptor α, adenosine monophosphate-activated protein kinase phosphorylation and adiponectin receptors and inhibition of glucose-6-phosphatase and 3-hydroxy-3-methylglutaryl-coenzyme A reductase may be involved in the anti-hypercholesterolemic and anti-hypertriglyceridemic effects of the compounds (Williamson, 2017 ▶; Haddad and Eid, 2017 ▶; Naveed et al., 2018 ▶). Further studies are needed to pinpoint the bioactive compounds and mechanisms responsible for the anti-hypertensive, anti-hyperglycemic and anti-dyslipidemic effects of the leaf extract of* V. arctostaphylos*.
